# Phenotypic map of porcine retinal ganglion cells

**Published:** 2013-04-16

**Authors:** Patricia Veiga-Crespo, Patricia del Río, Marcel Blindert, Marius Ueffing, Stefanie M. Hauck, Elena Vecino

**Affiliations:** 1 Department of Cellular Biology, Faculty of Medicine, University of the Basque Country, Leioa, UPV/EHU, Vizcaya, Spain; 2 Microbiology, Faculty of Sciences, University of Burgos, Burgos, Spain; 3 Department of Protein Science, Helmholtz Zentrum München, Neuherberg, Germany; 4 Center of Ophthalmology, University Medical Center, Tübingen, Germany

## Abstract

**Purpose:**

Porcine retina is an excellent model for studying diverse retinal processes and diseases. The morphologies of porcine retinal ganglion cells (RGCs) have, however, not yet been described comprehensively. The aim of the present study was to créate a classification of the RGCs using the 1, 1′-dioctadecyl-3,3,3′,3′-tetramethylindocarbocyanine perchlorate (DiI) tracing method.

**Methods:**

About 170 RGCs were retrogradely labeled by injecting DiI into the optic nerve of postmortem eyes and statistically analyzed by two different clustering methods: Ward’s algorithm and the K-means clustering. Major axis length of the soma, soma area size, and dendritic field area size were selected as main parameters for cluster classification.

**Results:**

RGC distribution in clusters was achieved according to their morphological parameters. It was feasible to combine both statistical methods, thereby obtaining a robust clustering distribution. Morphological analysis resulted in a classification of RGCs in three groups according to the soma size and dendritic field: A (large somas and large dendritic fields), B (medium to large somas and medium to large dendritic fields), C (medium to small somas and medium to small dendritic fields). Within groups, fine clustering defined several subgroups according to dendritic arborization and level of stratification. Additionally, cells stratifying in two different levels of the inner plexiform layer were observed within the clusters.

**Conclusions:**

This comprehensive study of RGC morphologies in the porcine retina provides fundamental knowledge about RGC cell types and provides a basis for functional studies toward selective RGC cell degeneration in retinal disorders.

## Introduction

Retinal ganglion cells (RGCs) constitute a diverse cell population decoding and transmitting the visual information through the optic nerve to the visual centers. The knowledge regarding RGC morphologies and dynamic functions reflecting their physiology is essential to understand the role of RGCs in retinal degenerations, such as glaucoma, retinal ischemia, and diabetic retinopathy. The neuronal plasticity of adult retina, even in adults, has been reported and documents the importance of knowing the arborization of dendritic fields in nonclinical and clinical manifestations. Recent advances provide functional assessments of visual changes in glaucoma patients correlated with the loss of visual field in the eye. An extensive reorganization of visual terminal area has also been detected in macular degeneration patients, and it is possible to observe alterations of geometry of retinal projections in a rat glaucoma model [1].

RGCs have been morphologically classified in a large number of species, including monkey [2], cat [3], rabbit [4], rat [5,6], and mouse [7-9]. The morphological criteria commonly employed to classify RGCs has been soma size and dendritic field dimensions. The dendritic trees of the RGCs determine the position, size, and shape of its receptive field [10]. In certain species, this approach has been validated by functional data demonstrating that different RGC classes project to different targets that control the visual functions [11,12].

It is now well established that RGCs comprise several classes with distinct anatomic and physiologic properties [3,11,13]. Two types of RGCs are the M and P cell classes from which signals are transmitted to the magnocellular and parvocellular layers, respectively, of the lateral geniculate nucleus in mammals. They respectively correspond to the anatomically identified parasol and midget retinal ganglion cells described by Polyak [14].

In the mouse, at least 11 RGC groups have been described based upon their morphology [9]. These groups have been specifically established by measuring the dendritic field size, branching pattern, and stratification within the inner plexiform layer (IPL). Morphological analyses were performed using diverse methods, including labeling by particle-mediated gene transfer, by transgenic expression of alkaline phosphatase [6,7], or by expression levels of parvalbumin [15]. In a study of the RGC in the rabbit retina [4], four methods were used to fill the RGCs (microinjection, particle-mediated insertion of gene coding for green fluorescent protein, particle-mediated introduction of 1, 1′-dioctadecyl-3,3,3′,3′ tetramethylindocarbocyanine perchlorate (DiI), and photofilling), and results from each method confirmed the findings. Although the frequency and clarity of a particular type of cell varied depending on the RGC markers used, most of the cells were independently revealed by each method [4].

The most common approach employed for cell staining is the use of lipophilic compounds. These compounds (DiI, 3,3′-dioctadecyloxacarbocyanine perchlorate [ DiO], 1,1′-dioctadecyl-3,3,3′,3′-tetramethylindodicarbocyanine perchlorate [DiD], 4-(4-(dihexadecylamino)styryl)-N-methylpyridinium iodide [DiA], and 1,1′-dioctadecyl-3,3,3′,3′-tetramethylindotricarbocyanine iodide [DiR]) have become a powerful alternative for the study of cell morphology and for demonstrating the anatomic relationships between different cell groups. Accordingly, this method has been used for the study of RGC morphology in several animals [4,6,9,16].

Among experimental animals, the pig has not yet been thoroughly studied with respect to RGC morphology [17]. This is despite the recognition that the porcine retina has a high resemblance to the human retina, which means it is an attractive nonprimate model for exploring preclinical efficacy of new pharmaceutical therapies for different human diseases [18-20]. The porcine retina is more similar to the human retina than to those of other large mammals, such as dogs, goats, and cows, and shares many similarities with those of humans [21]. Furthermore, its holangiotic vascularization of the retina is similar to that of humans [22]. Pig eye and retina resemble those of humans in size, number, and distribution of rods and cones, shape, and function. The porcine model comprises other important attributes, such as a well-characterized immune system and the applicability of tools used for diagnostics in ophthalmology, such as optical coherence tomography [23,24], corneal topography imaging, or multifocal electroretinogram, supporting the use of this animal as a good model for ophthalmological studies [25-27].

In previous studies we established three classes of RGCs based upon soma size (small, medium, and large) and mapped the correlation between their distribution and the topography of the porcine retina [20]. We further found that in vitro, RGCs are affected by the presence of different factors, including Müller cells that activate cell survival and neuritogenesis [28]. We previously characterized the relationship between RGC size and their sensitivity to damage in experimental glaucoma [25] and found that RGCs can change their morphological features under several pathologies. In glaucoma, some RGCs die selectively by apoptosis [29,30], resulting in blindness. However, the sensitivity of the different RGC morphologies to death is controversial. Diverse studies point toward a nonselective loss of cells in terms of RGC size [31]. It has been well documented that during experimental glaucoma, the retina exhibits a higher loss of RGCs in peripheral regions, including an increase in the mean soma area of surviving RGCs [25]. The ability of some RGCs to increase soma area size before death could either be associated with the necessity of covering more retinal space [25,32] or with the loss of osmotic regulation during apoptotic processes [33].

In the present study we analyzed porcine RGC morphological features by using dendritic field dimensions and the level of stratification in the IPL. We used two different statistical methods to create a comprehensive and reliable clustering of porcine RGCs.

## Methods

### Animal procedures

We analyzed 170 ganglion cells from nine retinas. Adult pigs used for the study (n=5) were between 3- and 6-months old. Porcine eyes were obtained immediately after death from the local slaughterhouse and transported in cold CO_2_-independent Dulbecco’s modified Eagle’s medium (DMEM; Gibco-Life Technologies, Madrid, Spain) supplemented with penicillin (10 U/ml) and streptomycin (10 μg/ml). The eyes were cleaned of all impurities (e.g., muscle, facia). All experimental methods and animal care procedures adhered to the Association for Research in Vision and Ophthalmology Statement for the Use of Animals in Ophthalmic and Vision Research and were approved by the University Institutional Animal Care and Use Committee.

### DiI labeling

The labeling method consisted of the application of lipophilic 1, 1´-dioctadecyl-3, 3, 3′, 3′-tetramethyl-indocarbocyanine perchlorate (DiI) (D-282; Molecular Probes; Life Technologies, Madrid, Spain) in the optic nerve. The optic nerve from the extracted eyes was then partially sectioned close to the globe, and a crystal of DiI was inserted following a transverse section to the nerve. The section was closed using a 5/0 surgical suture (VICRYL^®;^; Ethicon Endo-Surgery (Europe) GmbH, Norderstedt ,Germany) and one drop of 4% agarose. The eyeball was cut open around the ora serrata, the lens and vitreous were removed, and the posterior eyecups with the retina were immersed in 4% paraformaldehyde (in 0.1 M phosphate buffer) for approximately 6 months at room temperature in darkness. After 6 months the retina was dissected from the eyecup, mounted on slides, and coverslipped for image acquisition.

### Data analysis

Images were taken with a confocal microscope (Olympus FV 500; Olympus, Tokyo, Japan) from the mid periphery of the retina by using the optical disc (OD) as a reference point [20], using a 20× objective numerical aperture (NA 0.7), and a He-Ne laser (543 nm). Stacks of images (195 μm thickness) were taken from the innermost part of the cell body to the total extension of the IPL, including the most distant branches of the analyzed cell. The z-stack pictures were analyzed with the Olympus microscope software. The image processing was performed with a digital palette (Easypen, Genius, Taipei, Taiwan) with image-analysis software (Scion Image; Scion, Frederick, MD). These analyses determined the cell parameters: RGC soma size, major axis length dendritic field area size, dendritic arborization, and depth of stratification.

### Definition of the retinal ganglion cells analyzed parameters

To characterize RGC somas, we calculated the area size and the major axis length as follows. The soma area was defined by a line around the soma contour, and the soma major axis was defined by the distance between the two most distant points from the soma area [20]. Each RGC soma was outlined using the digital palette, and the data were transferred for subsequent statistical analysis. The major axis length and the area were calculated directly by the software.

The dendritic field area was calculated by creating a convex polygon matching all distant points from the dendrites using a graphic tablet as previously described [34]. Briefly, somal and dendritic field sizes were expressed as the diameter of a circle having the same area. The computer also calculated the parameter of the best-fitting ellipse to the dendritic field perimeter. The convex polygon was considered to be a solid shape of uniform thickness; the secondorder central moments of this shape were calculated and used to determine the parameters of an ellipse having the same central momments. The size of the ellipse was then scaled so that it had the same area as that of the convex polygon. The ellipse routines were calculated using the software ImageJ. The number of primary dendrites was defined by the number of dendrites connecting directly to the RGC body. The levels of stratification within the IPL were measured using three-dimensional reconstruction of each picture, and distances within the IPL were defined by taking the z-axis reading following the method of Sun et al. [6] and Coombs et al. [16].

To measure the thickness of the IPL and determine the stratification levels, the compilations of z-stacks were analyzed. The levels of stratification were expressed as a percentage of the thickness of the IPL, with 100% representing the boundary between the IPL and the ganglion cell layer (GCL) boundary and 0% representing the most distant part of the IPL from the ganglion cell layer. Thickness of the IPL from 4',6-diamidino-2-phenylindole (DAPI)-stained histological sections of the retina was determined after the measurements of the cells. A stratification range was calculated, representing the percentage interval between the innermost and outermost parts of their dendritic field [16].

### Statistical analysis

RGC measurements were statistically analyzed with the aim of distributing the cells in clusters based upon their morphological parameters. The statistical methods used Ward’s algorithm and k-means clustering with R statistical software (R software, R Foundation, Viena, Austria). We then determined the distribution of possible clusters by creating a dendrogram. We applied the Ward’s joining algorithm, which is a hierarchical procedure that determines the clustering of points in an N-dimensional parameter space. The algorithm first computed a matrix of squared Euclidean distances between every pair, which were joined in groups of two by using the minimum squared distance as criterion. Finally, all data points were linked in a single tree where the lengths of the branches represented the degree of relatedness of the groups. The number of clusters was determined by applying a cut-off valued for the minimal branch length between clusters. Application of the algorithm was followed by the k-means algorithm, which is a nonhierarchical method for clustering points in an N-dimensional space that requires a predetermination of the number of clusters. In this work, the number of clusters was determined by applying the Ward’s algorithm.

The K-means algorithm assigned each point to a cluster and then refined that assignment by iterative application of the criterion in which the squared Euclidean distance between each case and the centroid of the cluster to which it belonged had to be smaller than the corresponding distance to the centroids of different clusters. The data were normalized to a mean of 0 and a standard deviation (SD) of 1. The cell area, major axis, and dendritic field area were taken as the main clustering parameters. The K-means for the different number of clusters (3 to 9) were calculated.

To establish the quality of the clusters, the Silhouette value *S(i)* was calculated. In this approach each cluster had a silhouette, the value of which showed which values were inside the clusters and which were merely between clusters. The average silhouette width provided an evaluation of clustering validity and was used to select an appropriate number of clusters [35].

For the *S(i)* calculation, the data needed to be grouped by a previous clustering technique, such as Ward’s algorithm and the k-means used in this work, to obtain *k* clusters. For each datum *i*, it was necessary to calculate the value of the average dissimilarity of *i* within the cluster *a(i);* the smaller the value of *a(i),* the better the matching inside the cluster. The value of *b(i)* represented the average dissimilarity of *i* with the data of another single cluster. The cluster with the lowest average dissimilarity to *i* was the neighbor cluster. Therefore, *S(i)=*[*b(i)–a(i)*]*/maximum*[*a(i),b(i)*] [35]. According to this equation, the *S(i)* value of the entire data measured the appropriateness of the data that had been clustered.

The principal component analysis (PCA) was calculated to create a cluster plot. This involved a mathematical procedure that transformed several possibly correlated variables into a smaller number of uncorrelated variables called principal components. The first principal component accounted for as much variability as possible, and each succeeding component accounted for as much of the remaining variability as possible.

## Results

Previous studies on porcine retina demonstrated the existence of three RGC groups based on their different soma size in vivo [20] and in vitro [21]. In the present study, additional parameters, such as dendritic field size and stratification level, were analyzed to advance from a topographical to a detailed morphological classification. The morphological description of RGCs enabled us to classify the cells in different clusters in an organized structure based on statistical analysis.

### Selection of the parameters and number of clusters

Soma area size, major axis length, and dendritic field area size were the selected parameters to create the clusters from the total of the RGC population. Additional parameters related to particular aspects of dendritic arborization and depth of stratification let to carry out the classification of RGCs in subclusters.

**Figure 1:**
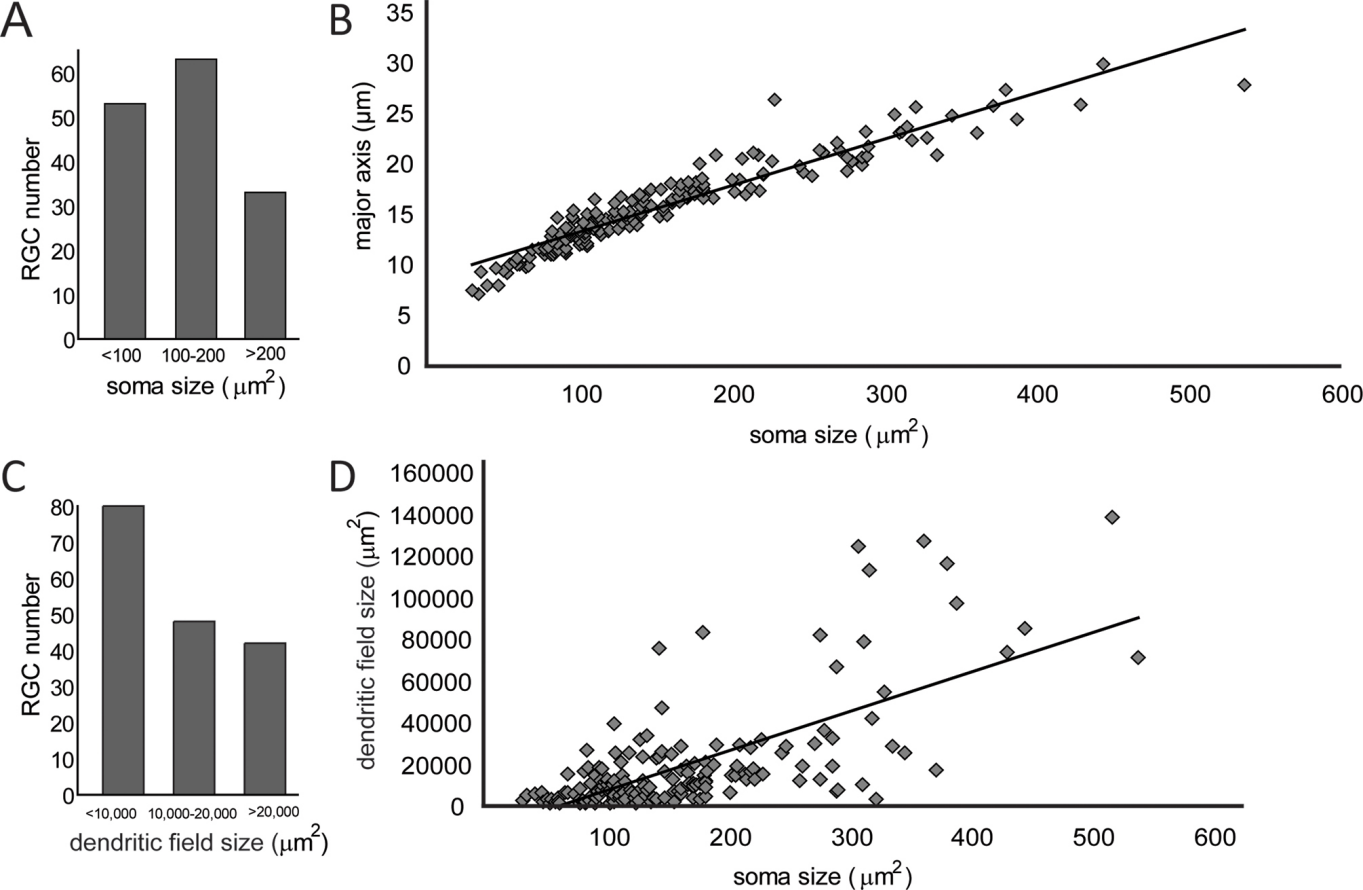
Distribution of retinal ganglion cells in porcine retina with respect to their soma area sizes and dendritic field sizes. The retinal ganglion cells (RGCs) from nine retinas were measured for three different parameters: soma major axis length, soma area size and dendritic field area size. Cells were grouped into three different groups according to (**A**) soma area: <100 μm^2^, 100–200 μm^2^, >200 μm^2^ as well as (**C**) dendritic field area (<10,000 μm^2^, 10,000–20,000 μm^2^, >20,000 μm^2^. Major axis length and the soma area sizes of RGCs correlate linearly (**B**), whereas soma area 1 sizes do not correlate linearly with dendritic field area sizes (**D**).

In this study RGCs were grouped with respect to their soma area size as small (<100 µm^2^), medium (between 100 and 200 µm^2^), and large (>200 µm^2^) and to their dendritic field area size as small (<10,000 µm^2^), medium (between 10,000 and 20,000 µm^2^), and large (>20,000 µm^2^). We then investigated whether major axis length and dendritic field area size correlated to soma area size. We demonstrated that while major axis length directly correlated to soma area size, dendritic field area size did not linearly correlate to soma area size (Figure 1).

**Figure 2:**
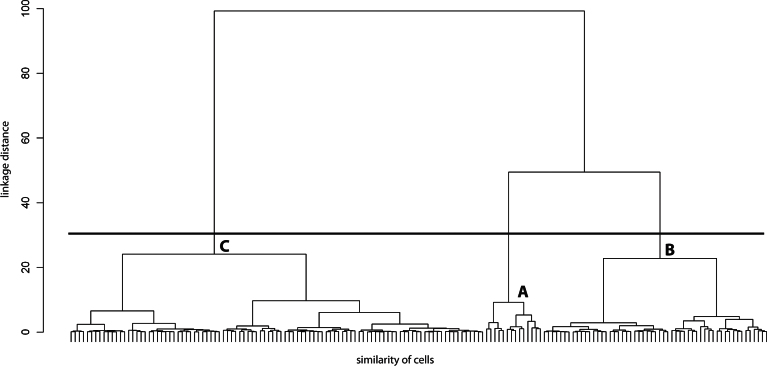
Ward’s dendrogram for retinal ganglion cells. The Ward’s method showed that retinal ganglion cells (RGCs) could be differentiated into three clusters (A, B, C). The obtained dendrogram for cluster analysis is represented here. The relative similarity of cells (x-axis) was shown in the linkage distance (y-axis) for all RGCs analyzed (n=170). The cluster origin for the cluster obtained in this work was designated with the corresponding letter. A continuous 3 pt-weight line divides the three main clusters in the study.

**Figure 3:**
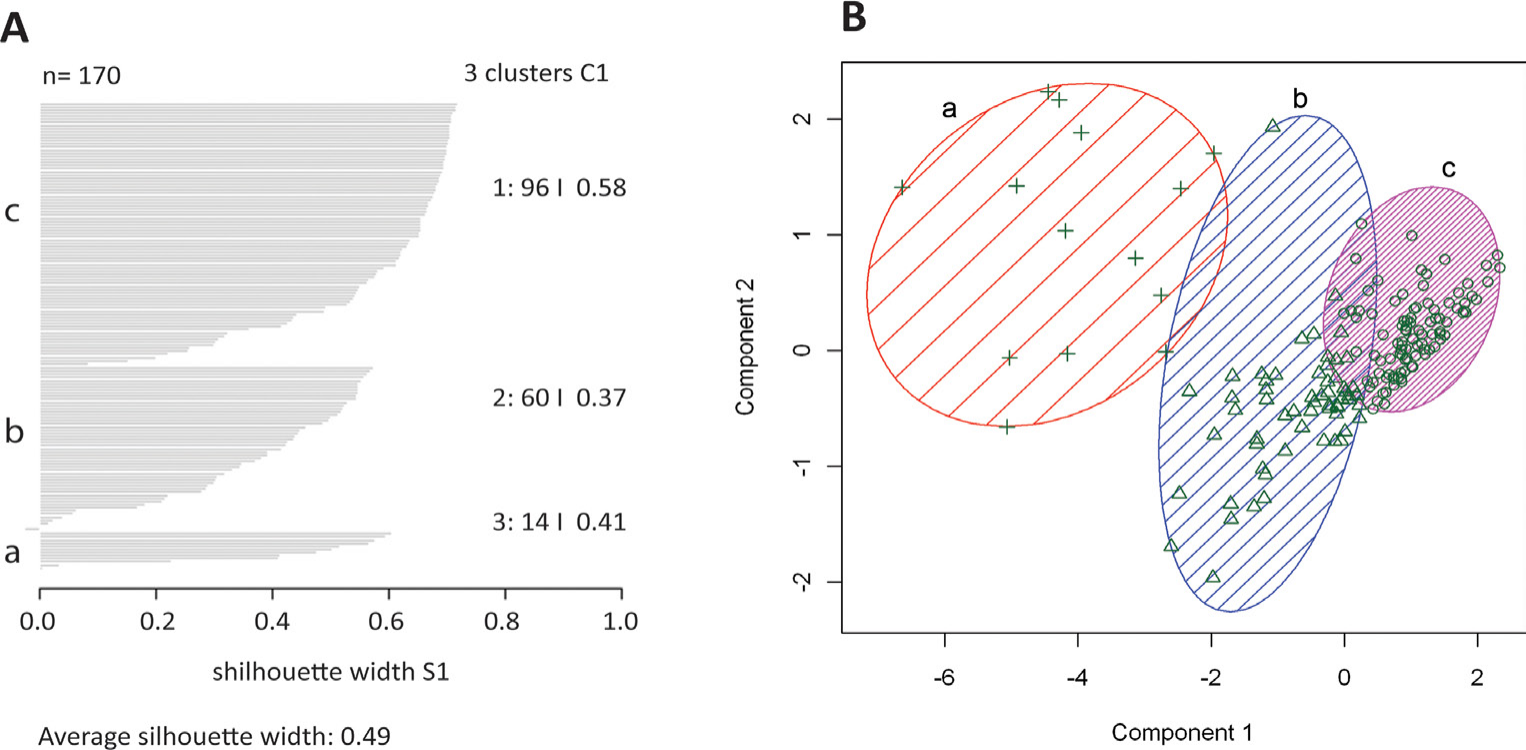
K-means analysis of the diverse porcine retinal ganglion cell population. **A**: The Silhouette plot was elaborated according the best clustering distribution, taking into account the given parameters of soma major axis length, soma area size, and dendritic field area size for all retinal ganglion cells. The number of individuals in each cluster is defined and the mean value of the silhouette plot (0.49) is also indicate (where n is the sampling size and represent the total Number of analyzed cells; a, b, and c represent the three obtained clusters; the cluster a contains 96 cells; the cluster b 60 and the cluster c 14). **B**: The representation of the principal analysis components was carried out. The representative clustering plot for all retinal ganglion cells showed their distribution into three major clusters (+: cells into cluster a; Δ: cells into cluster b; ¢′: cells into cluster c).

Ward’s method was initially applied to obtain a clear separation of clusters in the resulting dendrogram (Figure 2). Once the appropriate number of clusters was determined, the clusters were depicted in the dendrogram for the whole population of labeled RGC cells (Figure 2). The k-mean analysis was performed using different numbers of possible clusters (from 3 to 9, data not shown), and the silhouette plots were compared to select the silhouette number closest to 1 for a perfectly clustered point. Among the nine clustering possibilities tested, the highest silhouette width appeared with three clusters (average silhouette value 0.49; Figure 3A). It was evident in the cluster plot that among the three clusters represented, each one had members that differed from others and did not interconnect with the members of the other two clusters, thus exhibiting a distinct separation. However, the other two subclusters were interconnected and contained members that had overlapping parameter values.

To create a graphical display of the clusters, we used the CLUSPLOT algorithm of Pison et al. [36], which describes the objects with their interrelations and at the same time illustrates the clusters. The objects are represented as points in a bivariate plot and the clusters as ellipses of various sizes and shapes as well as their relative position. The dimension of the data are reduced by principal component analysis (PCA), which yields a first component with maximal variance (component 1, Figure 3B), then a second component with maximal variance among all components orthogonal to the first (component 2, Figure 3B). The principal components lie in the directions of the eigenvectors of a scatter matrix. The resulting graphic shows the first two principal components and lists the percentage of the total variance explained by them. In contrast to an individual PCA analysis where the components indicate which variables can be combined to explain the data, the PCA in CLUSPLOT is only used as a dimension reduction technique. With the CLUSPLOT function, it is possible to identify the effectiveness of clustering. In the case of successful clustering, the clusters are clearly separated in the principal plane. The main characteristics of subclusters are summarized in Table 1.

**Table 1:** Clusters of pRGCs.

pRGC cluster	Soma area/ µm^2^	Dendritic field area/ µm^2^	N of cells/ cluster
A1	410±81	105,038±24,383	9
A2	241±74	77,210±6,594	5
B1	276±49	25,530±10,542	23
B2	191±28	15,941±5,232	23
B3	176±11	6,838±2,503	10
C1	120±20	19,312±8,133	25
C2	122±19	5,056±2,507	35
C3	89±12	15,773±5028	8
C4	73±21	3,911±2,218	32

### Cluster A—large retinal ganglion cells with large dendritic fields

**Figure 4:**
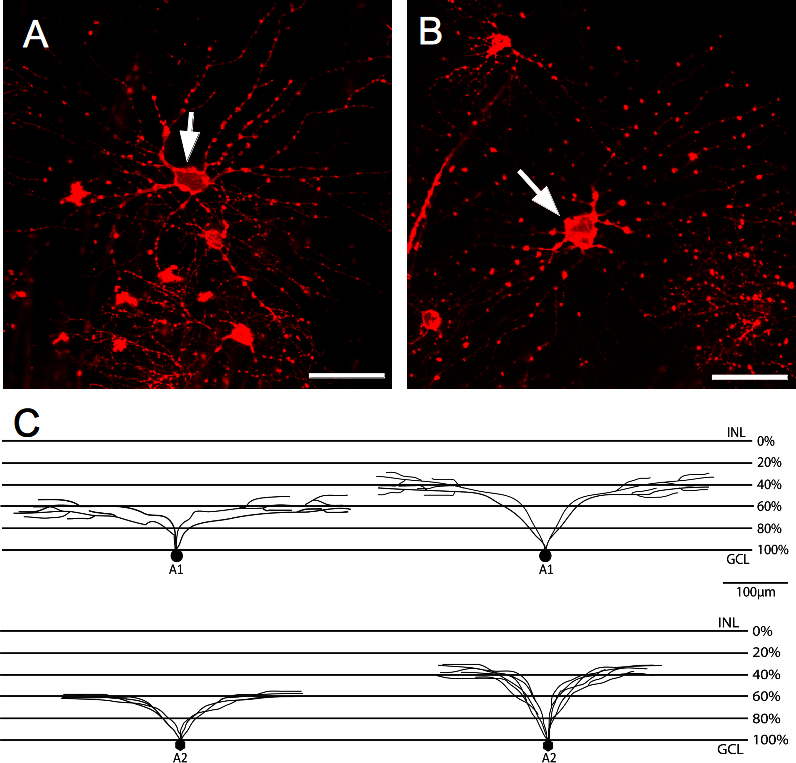
Representative pictures from the A cluster. **A**: The A1 subcluster contains cells with the largest somas and dendritic fields from the total population. **B**: The cells contained in the A2 subcluster display large somas and large dendritic fields. Scale bars are 50 µm. Arrows indicate the cell belonging to the specific subcluster. **C**: Schematic representation of retinal ganglion cells showing the possible branching and levels of stratification for the subclusters. INL is the inner nuclear layer; GCL is the ganglion cell layer.

The largest somas and dendritic fields found among all classified RGCs were the cells contained in cluster A (Figure 4). A total of 14 RGCs were associated with this cluster. They had, in general, large somas (>200 µm^2^) and large dendritic fields (>20,000 µm^2^). This cluster was divided into two different subclusters, A1 and A2.

In subcluster A1 (Figure 4A), soma area sizes were the largest in the population (410±81 µm^2^) and they had the largest dendritic field areas (105,038±24,383 µm^2^). This subgroup was denominated as the giant RGCs of the porcine retina. These cells had a homogeneous round cell body with four to seven primary dendrites that emerged from the large soma. The dendrites branched in a wider Y-shaped manner, with a greater distance between branching points that gave a comparatively sparse appearance to the dendritic tree. About 63±10% of these cells stratified in the IPL, while the 43±3% stratified in the outer part of the IPL (Figure 4C).

A2 cells had a similar morphology as A1 cells except that their mean soma area was smaller (241±74 µm^2^) and had a polygonal shape (Figure 4B). The mean dendritic field area was 77,210±6,594 µm^2^ with five to seven primary dendrites emerging from the soma and stratifying either at 33±9% of the IPL or at 59±0.03% of the IPL (Figure 4C).

### Cluster B—medium to large retinal ganglion cells with medium-sized dendritic fields

**Figure 5:**
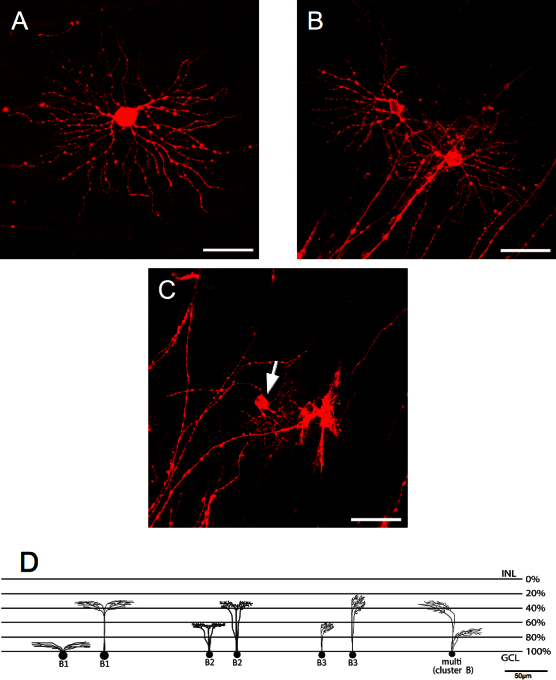
Representative pictures of the B cluster cells. Retinal ganglion cells (RGCs) representing the subclusters are shown. **A**: B1 subcluster contains cells with large soma areas and medium to large dendritic fields. **B**: B2 subcluster contains cells with medium soma areas and medium dendritic fields. **C**: B3 subcluster contains cells with medium soma areas and small dendritic fields. The scale bar is 50 µm. Arrows indicate the cell belonging to the specific subcluster. **D**: This is a schematic representation of RGCs showing the possible branching and levels of stratification for the subclusters. INL is the inner nuclear layer; GCL is the ganglion cell layer.

RGCs with medium to large somas and medium-sized dendritic fields represented the cells from cluster B (Figure 5). The 56 cells that belonged to this cluster were divided into three subclusters. Subcluster B1 (Figure 5A) contained cells with large somas (276±49 µm^2^) and medium to large dendritic fields (25,530±10,542 µm^2^). The number of primary dendrites was between four and six, and the stratification pattern was highly variable, reaching minimally to 36±9% of the IPL to a maximum 89±11% of the IPL (Figure 5D). The main morphological feature of this subcluster was a large soma with a homogeneous round form. The dendritic field followed a radial branching pattern in most cases, with equal distance of separation between the main dendrites situated homogeneously around the soma and secondary branches showing many protrusions and spines.

Subcluster B2 (Figure 5B) had medium to large somas (191±28 µm^2^) and medium dendritic fields (16,941±5,232 µm^2^). Their morphology was similar to B1 cells but had smaller dendritic fields. Their primary dendrites (four to six) were thick and tapered from soma to periphery; they branched frequently along their course and exhibited a nonlinear shape. The dendritic field contained many short dendritic branches. The distal dendrites terminated in a y-shaped pattern. Subcluster B2 stratified in the IPL at 37.5±1.5% and at 66±1% (Figure 5D).

Subcluster B3 (Figure 5C) had mean soma areas of 176±11µm^2^ and had small dendritic fields (6,838±2,503 µm^2^) as compared to their medium somas, which had three to six primary dendrites. B3 dendritic arbors could be stratified as a B2 subcluster in the IPL at 37.5±1.5% and at 66±1% (Figure 5D).

The resemblance between B2 and B3 cells was based on their curly and dense arborization pattern with tiny and fine dendrites. The main difference between these cells was the sideward orientation of B2 cells compared to B3 cells.

### Cluster C—medium to small retinal ganglion cells with medium-sized dendritic fields

**Figure 6:**
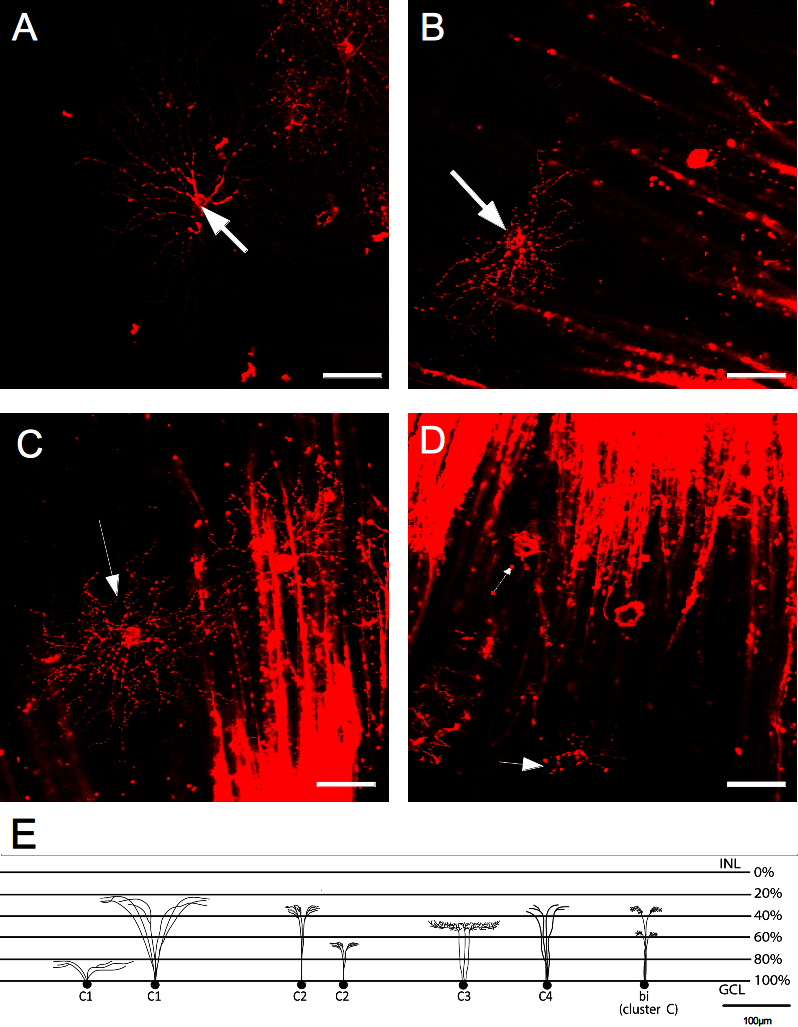
Cluster C cells containing medium to small soma areas and medium to small dendritic fields. **A**: C1 subcluster contains cells with medium to small soma size and large to medium dendritic fields. **B**: C2 subcluster contains cells with medium soma sizes and small dendritic fields. **C**: C3 subcluster contains cells with small soma size and medium dendritic fields. **D**: C4 subcluster contains the smallest retinal ganglion cells (RGCs) with small soma size and small dendritic fields. **E**: This is a schematic representation of RGCs showing the possible branching and levels of stratification for the subclusters. INL is the inner nuclear layer; GCL is the ganglion cell layer.

The 100 RGCs representing cluster C created the most heterogeneous group within the defined clusters, showing medium to small-sized somas and medium-sized dendritic fields (Figure 6). This large cluster was divided into four subclusters.

The cells belonging to subcluster C1 (Figure 6A) had a general medium to small soma size and exhibited large to medium dendritic fields. Soma areas in this subcluster had a mean value of 120±20 µm^2^ and dendritic fields of 19,312±8,133 µm^2^ with three to eight primary dendrites emerging from the soma. In general, C1 members stratified in the IPL at depths of 33±9%, but some cells with large dendritic fields stratified at 83±11% (Figure 6E). Subcluster C2 (Figure 6B) was constituted by cells with medium soma sizes 122±19 µm^2^, small dendritic fields, four to six primary dendrites, and a mean dendritic field area of 5,056±2,057 µm^2^ that stratified at 35±10% and 67±12% of the IPL level (Figure 6E).

In subcluster C3 (Figure 6C), the mean soma area was 89±12 µm^2^ and the mean dendritic field area was 15,773±5028 µm^2^. This subcluster exhibited the same number of primary dendrites as the other subclusters within this group, with stratification levels of 47±12% in the IPL (Figure 6E).

Subcluster C4 (Figure 6D) comprised the smallest RGCs in the study and the most variable set of cells. The soma area had a mean of 73±21 µm^2^ and the mean dendritic field area was 3,911±2,218 µm^2^. Therefore, they were defined as RGCs with small somas and a small dendritic field. Most of cells in this subcluster stratified at 32±8% level of the IPL and sprouted three to eight primary dendrites (Figure 6E).

### Bistratified and multistratified cells

Among all RGCs analyzed, it was possible to localize cells that stratified in two different levels of the IPL. Cells were called multistratified when one dendritic field stratified in two different levels of the IPL and were called bistratified when two different dendritic fields stratified in two different IPL levels. These cells were distributed in subclusters according to their soma and dendritic field dimensions. All multistratified members were found in cluster B (Figure 5D), whereas all bistratified cells were found in cluster C (Figure 6E). The present results confirmed that it was possible to group the porcine RGCs in several clusters sharing coincidental features, while other features diverged within each other.

## Discussion

**Figure 7:**
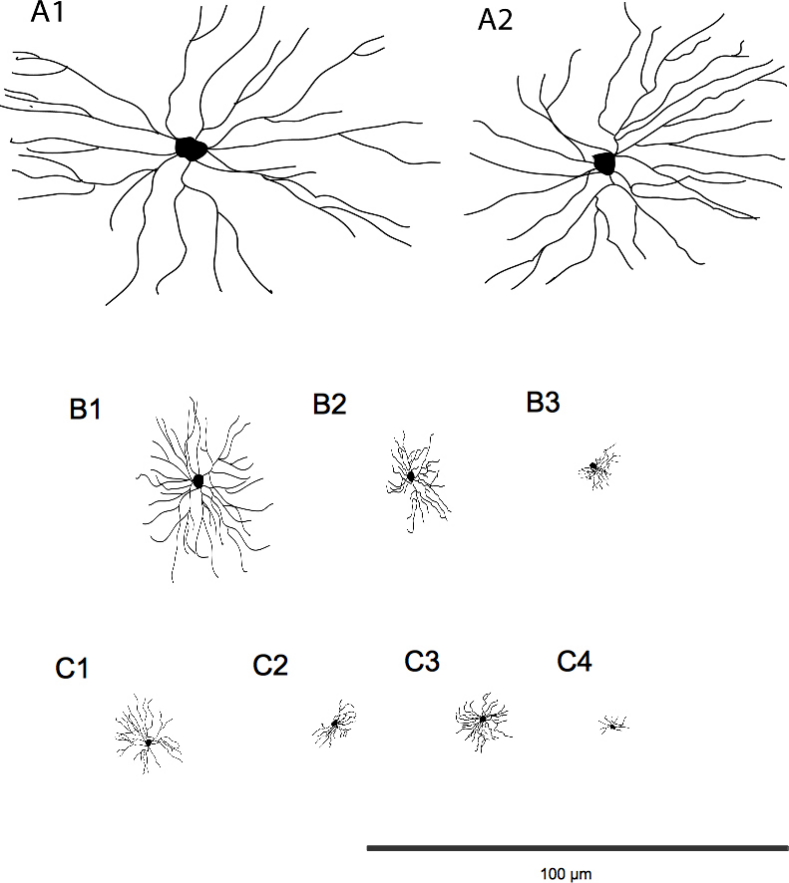
Schematic diagrams of the representative models of retinal ganglion cells for the different subclusters noted above. The differences between the dendritic fields along the different subclusters can be observed. The scale bar is 100 µm.

In previous studies the topology of RGC density in relation to soma size distribution in the porcine retina was characterized [20]. In the present study analysis was extended to the porcine RGC phenotypes by including dendritic field dimensions and the level of stratification in the IPL as additional parameters. This population study resulted in the creation of three clusters, which were then subdivided into nine different subclusters (Figure 7).

### DiI as a tracing method for retinal ganglion cells

Among many methods employed to trace RGCs, the introduction of DiI particles has been the most recommended method due to their striking and precise filling of cells with excellent dendritic details [4,37-39]. In comparison to other methods, such as photofilling and lucifer yellow, where the high brightness gradient was a disadvantage when tracing the smallest somas, DiI appears to be more uniform, providing excellent visualization of the cell. For the present study of porcine RGC morphology, we labeled the RGCs exclusively with DiI and found that this tracer was an optimal tool to discern the specific contours of the soma and the details of dendritic fields. Since our clustering analysis was based specifically on soma area size, major axis length, and dendritic field area size, it was essential to include only those cells with completely labeled dendrites. The criterion for complete filling was the distinct ending of a dendrite, which often terminated in a tiny enlargement or lobule. It was possible to observe complete labeling for nearly all detected RGCs, which were then included in the present study without biased preselection.

The disadvantage of this labeling approach was that most labeled cells that were traced and analyzed were located in the mid-peripheral part of the retina. One of the disadvantages of DiI came from its timing-depending manner. We found that 6 months of incubation with crystals of DiI was the optimum time for tracing the contour of the soma and the details of the dendritic field in the mid peripheria. However, if the time of incubation was longer, the resulting background was too high, and it was impossible to detect the RGC contours. Therefore, we obtained an unexpectedly higher number of medium to small RGCs as compared to the number of large RGCs. Since the majority of large RGCs are localized in the porcine retinal periphery [20], it would be necessary to investigate the peripheral part of the retina to discern the existence of RGC types that may not belong to the identified clusters. Despite this limitation, we concluded that the use of DiI as a tracing method was valuable to define the finest details of RGC morphology and evaluate the differences in relative sizes, an important value for cell classification. Since the diverse population in the mid-peripheral retina was comprehensively analyzed, it would be expected that the relative sizes across the whole retina were similar. However, additional methods should be used to complement the dendritic architecture to their physiologic properties.

### Features of retinal ganglion cell groups

While the correlation between major axis length and soma area size proved to be linear, soma area sizes and dendritic field area sizes showed no linear correlation. Some cells had large somas and large dendritic fields (clusters A and B), whereas other cells had small somas and medium dendritic fields (subcluster C3). The soma and dendritic field sizes were more consistent and proportional within the cluster of large RGCs than for medium or small RGCs.

The present results are in accordance with studies in the mouse [40], where the relation between soma area size and dendritic field size were not correlated. In mouse, the soma sizes overlapped among the groups, while dendritic field sizes were different for cell types. Two different statistical methods were designed to create an unbiased cluster classification. A nonhierarchical K-means clustering method was used in conjunction with the hierarchical Ward’s joining method. The hierarchical method has been broadly used for clustering studies [8,16] but did not give exact information regarding the optimal number of clusters. There were several advantages to using a nonhierarchical method like the K-means instead of a hierarchical method to ensure the most appropriate number of clusters in a given population. The K-means enabled the degree of clustering in the data to be evaluated. The most adequate clustering number was decided by selecting the highest silhouette plot. This method could then combine the information from the two statistical methods used, obtaining the most adequate number of clusters from the K-means study and selecting and describing the groups from the Ward’s dendrogram classification. The selected main parameters allowed generating a complete statistical study where a total number of three main clusters were obtained.

Once the number of clusters was determined, other additional parameters (number of primary dendrites, arborization pattern, and stratification level in the IPL) were used to create subclusters related to additional phenotypic observations [41]. The actual rules for RGC classification hold that each type of retinal ganglion cell will have: a) unique receptive field properties; b) a distinct dendritic tree morphology, viewed in a retinal whole mount; and c) a unique pattern of stratification in the retina’s IPL [42]. All these could be observed in the analyzed cells and in their division into the different clusters in this work.

The clusters were identified by decreasing soma size areas. Cluster A was formed by two subclusters A1 and A2, where the RGCs presented the largest somas and dendritic fields of the total population (Figure 7A1 and A2). The morphological features of this cluster comprised rounded and homogeneous somas for the A1 subcluster and heterogeneous somas for the A2. The number of dendrites varied slightly, presenting a large number of primary dendrites but fewer secondary dendrites for A2. Cluster A resembled comparative clusters in other animals, such as the RG_A1_ in mouse [6], the G11 in rabbit [4], and the gamma cells in monkey [34]. Cluster A appears to be a paramorphic pair, which could be the ON and OFF alpha cells described in previous studies [4,9]. For the largest RGCs, dendritic field areas were smaller than in comparative clusters in rabbits [4]. However, since special care was taken to only measure dendritic field sizes of cells that appeared completely labeled, the observed discrepancy could be due to either a species difference or uncertainties in separation of neighboring dendritic fields. In these cases, an average total dendritic field area could only be measured approximately.

The RGCs in cluster B appeared to have medium to large somas and medium dendritic fields, progressively smaller from subgroup B1 to B3 (Figure 7B1, B2, and B3). The rounded somas and homogeneous dendritic fields of B1 were comparable to their equivalent G10 in rabbit [4] and RG_B1_ and M4 in mouse retina [6,9]. These cells clearly seem the ON delta cells previously defined, which are project selectively to the medial terminal nucleus of the accessory optic system, being probably the ON-type directionally selective cell [4]. B2 cells could correspond to the RG_B4_ group in mouse; however, they displayed larger dendritic field dimensions [6]. Cluster B3 resembled the comet-like shape of RG_C6_ [5] and M5a in the mouse retina [9], indicating that these cells could be related and only stratified in the OFF sublamina [6]. The observed side-oriented form of their dendritic fields could indicate a conserved cell function in different species. Cluster C (Figure 7C1, C2, C3, and C4) was heterogeneous, presenting small soma sizes and medium dendritic fields accounting for several specific features within the subgroups.

The rhomboidal form and the smooth long primary dendrites from most members in C1 appeared to be similar to RG_C1_ and M9 in mouse retina [6,9]. According to the stratification depths in the IPL and the arborization of the dendritic fields, the cells from subcluster C1 could also be compared to G5 of rabbit [4]; based on their dendritic arborization in the IPL, these must be OFF cells. Subclusters C2 and C3 showed similarities in terms of their dendritic morphology but they diverged in orientation and size of these dendrites. They may share some similarities with RG_D1_ and M13, M14 cells in mouse, so it would not be unreasonable to assume that they were a type of ON–OFF nondirectional-selective cells [6,9]. The smallest RGCs grouping in subcluster C4 were difficult to compare with other studies. The G1 cluster in rabbit [4] could be the most comparable example when comparing soma area size and the stratification level. The ﬂattened “y” feature of the dendritic arbors of C1 and G1 cells identified them as local edge detectors [5].

B and C clusters have bistratified RGCs that could also be considered ON–OFF RGCs. As noted by Chalupa and Günhan [43], there is no clear boundary within the IPL separating the sublaminas in which RGCs send their projections. Some investigators have considered the inner three-fifths of the IPL as ON and the outer two-fifths as OFF [43]. However, the thickness of the IPL is not uniform among animals of a given species, nor it is uniform across the retina of a given animal. The same authors concluded that there is a strict correlation in the retina between structure and function. To our knowledge no electrophysiological studies have been performed in pig RGCs, thus we are unable to correlate the morphology and function of the cells described in the present study. In mice, however, a detailed study has correlated the morphological and physiologic parameters, concluding that mouse RGCs vary in morphology and physiology.

We presented here a detailed descriptive morphological classification of RGCs based on a large and properly stained population in the retinal mid periphery. This study will serve as a reference for understanding functions of distinct RGC subpopulations and will also provide a basis for understanding the morphological changes observed in the RGC population of porcine models in response to retinal degeneration.
